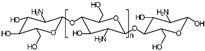# Research Status and Potential Direction for Thermoplastic Shape Memory Polymers and Composites: A Review

**DOI:** 10.3390/polym17101360

**Published:** 2025-05-15

**Authors:** Shuai Yang, Yang He, Zijian Song, Yingchun Li

**Affiliations:** 1 School of Materials Science and Engineering, North University of China, Taiyuan 030051, China; yangshuai@nuc.edu.cn (S.Y.); songzijian@nuc.edu.cn (Z.S.); 2 Shanxi Center of Technology Innovation for Polyamide Materials, North University of China, Taiyuan 030051, China; 3 Center for Composites Materials and Structures, Harbin Institute of Technology, Harbin 150080, China; he2007yang@163.com

**Keywords:** thermoplastic composites, shape memory polymers, applications, developing directions

## Abstract

Shape memory polymers (SMPs), due to the programmable deformation and recovery ability, exhibit widespread potential in fields of biomedical devices, smart actuators, and engineering structures. Thermoplastic SMPs, which possess the intrinsic linear molecular chain structures, are able to be processed through diverse methods, in addition to being re-processed after process-forming, compared with thermoset SMPs. The environmental recycling characteristics for thermoplastic SMPs describe their wide use potential and prospect. In this paper, a comprehensive description of mechanism, matrix polymers, actuations, and applications for thermoplastic SMPs and composites was reviewed. Furthermore, two promising potential developing directions, 4D printing metamaterial and dynamic covalent networks, were proposed. The multifunctionality and enhanced performances of thermoplastic SMPs and composites exhibited excellent application value, which is significant for future advancements.

## 1. Introduction

In nature, animals, plants, and microorganisms are able to perceive external stimuli, and make the corresponding response for adapting to the environment. Chameleons can change their own color to reflect changes in the surrounding environment for camouflage and protection. Sunflowers always face the sun to obtain more sunlight. Inspired by nature, researchers design and fabricate smart responsive materials, which could receive external stimuli, analyze and process the information, and make the corresponding response. Stimulus-responsive materials, according to the intrinsic characteristic, are divided into stimulus-responsive polymers and stimulus-responsive alloys. In 1932, Olander Sweden [1] firstly found the shape memory material of gold cadmium alloy, which could spontaneously recover to the original shape while heating to the threshold temperature. It is the first exploration into shape memory materials for human beings that the shape recovery behaviors of memory alloy. In 1950s, Charles [2] observed the shape memory behaviors of crosslinking polyethylene by accident, which opened the door for people to use shape memory polymers (SMPs). In the 1960s, SMP-based commercial products of thermal shrinkable polyethylene pipes were firstly produced, which is a milestone for the development of SMPs. Nevertheless, there was no defined concept for SMPs at that time. After the 1990s, the development of SMPs efficiently increased, where the matrix materials have been enriched, and the applications have been largely extended [3,4,5,6].

As a kind of smart stimuli-responsive material, SMPs are able to perceive external stimuli and information and make the corresponding response. These smart response characteristics including self-adapting, self-regulating, and self-healing upon the stimuli have attracted researchers’ attention, promoting the designing, manufacturing, and use of smart materials. In addition, the development of SMPs is not confined to the conventional single stimulus response, simple deformation manipulations, and narrow applications. As the development of science and technology continues, the multi-stimuli-responsive and multifunctional multi-SMPs with better performance have been researched in-depth and rapidly developed, which occupies a significant position in fields of aerospace structures, biomedical applications, and smart devices. When receiving external stimuli, SMPs can be deformed by the external force, and keep a stable temporary shape after the stimuli are done. Once receiving stimuli again, SMPs can spontaneously recover to their original shape. This fixity ability for temporary shape and recovery ability for permanent shape impart SMPs excellent programmable deformation abilities [7,8]. In addition, SMPs possess intrinsic advantages which do not exist in conventional materials, such as light weight, easy processing, low cost, and large deformation [9]. These excellent performances endow SMPs with wide application potential and value in fields of space deformable structures, biomedical tissue stents, and flexible smart sensors [10,11,12]. Conventional SMPs are almost heat-triggered SMPs, and the responsive stimulus originated from the increasing temperature of the external environment. While the environment temperature reaches up to the transition temperature, the phase transition of SMPs’ internal structures occurs, releasing the internal stress previously stored, and as a result, macroscopic shape recovery behaviors are triggered. At present, the external stimuli, which could be the actuation source for SMPs, have become multiplex, including heat, electricity, magnetism, light, solvent, and microwave [13,14,15], and the matrix polymers for SMPs have also been largely developed, including polyurethane [16,17,18], polylactic acid [19], polycaprolactone [20,21], polystyrene [22], polyimide [23,24], etc. Due to the unique stimuli-response and deformation recovery ability, SMPs exhibit wide potential and value. Nevertheless, commonly used SMPs are based in thermoset polymers now, due to the low cost and easy processing. The reviews and presentations for thermoplastic SMPs are relatively fewer. In this paper, we describe the mechanism, matrix polymers, actuations and applications for thermoplastic SMPs and the composites. Furthermore, two promising potential developing directions, 4D printing metamaterial and dynamic covalent networks, were provided and discussed.

## 2. Thermoplastic SMPs

**Figure 1:**
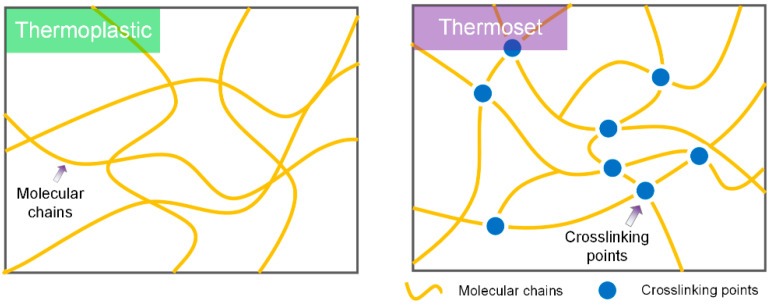
The internal molecular chain structures of thermoplastic and thermoset SMPs.

Thermoplastic polymers possess linear molecular chain structures in matrix, which are different from the crosslinking structures of thermoset polymers, as shown in Figure 1. Thus, they exhibit the apparent phase transition behaviors (glass–rubber transition or rubber-viscous flow transition) while heating above the glass transition temperature (T_g_) or melting point (lower than their decomposition temperature) [25]. Meanwhile, thermoplastic polymers have better processing performances compare with thermoset polymers. Due to the non-crosslinking linear structures, thermoplastic polymers are bound with intermolecular interactions such as Vander Waals interactions and hydrogen bonds [26] for the stabilization of whole materials. Thus, the phase transition and variable rigidity are the determining factors for shape memory behaviors of thermoplastic SMPs, and the phase transition temperature points could act as the switching points which trigger the shape memory behaviors.

Generally, there are two components in SMPs system for the operation of shape memory behaviors: permanent phases and reversible phases. The former are bound for the stabilization and recovery of permanent shape, which usually contain crystalline phases, rigid segments, and crosslinking networks. The latter ensure the deformation and fixity of temporary shape, which include amorphous phases and flexible segments. Nevertheless, there are no crosslinking networks in thermoplastic SMPs matrix, and the substitute for permanent phases are molecular chain entanglements. Based on the molecular structures, there are two different parts in thermoplastic SMPs matrix: stabilization phases and reversible phases. The former ensures the stability of molecular chains upon the external stimuli, which include crystalline phases, chain entanglements, and physical crosslinking networks, such as hydrogen bonds. The latter could be deformed and fixed, which mainly include amorphous phases and flexible segments. In addition, the reversible transitions in thermoplastic SMPs matrix might include crystalline/melting transition, glass–rubber state transition, and cracking–recombination of non-covalent bonds [27,28,29]. While heating above the transition temperature, phase transition occurs, and the temporary shape can be obtained via the external force. After cooling down, the molecular chains are frozen, and the produced internal stress is locked in the system. Heating the SMPs again, the frozen chains become active and release the internal stored stress, inducing shape recovery. Different from the thermosets, thermoplastic SMPs exhibit various processing techniques including hot pressing, melt extrusion, injecting molding, and addictive manufacturing, in addition to sustainable cycle practicability, which are the dominant superiorities and attract attention [30,31,32].

**Figure 2:**
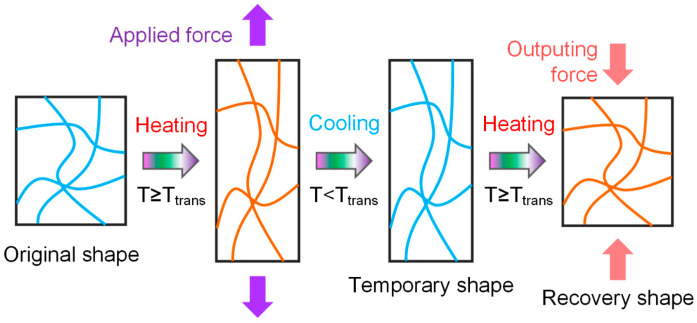
Shape memory behaviors of heat-triggered thermoplastic SMPs.

At present, heat-triggered thermoplastic SMPs could be the most widely used; the shape memory process is depicted in Figure 2. The prepared SMP sample is defined as the original shape. While heating the temperature up to the transition temperature, the temporary shape could be obtained by the external force. Maintain the temporary shape and cool down for shape fixity. Afterwards, heat the sample again, which could spontaneously recover to the original shape.

**Figure 3:**
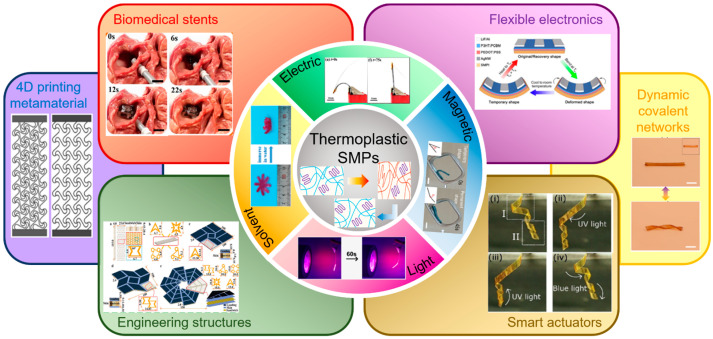
Diverse stimuli actuations, applications, and future directions of thermoplastic SMPs.

According to the different stimuli, thermoplastic SMPs could be divided into heat-triggered SMPs, electric-triggered SMPs, magnetic-triggered SMPs, light-triggered SMPs, and solvent-triggered SMPs [33,34,35]. In addition, due to advanced multifunctionalities, thermoplastic SMPs exhibit exceptional multi-stimuli responsiveness and multi-shape deformation abilities upon programmable manipulations [36,37]. Therefore, thermoplastic SMPs exhibit wide applications in fields of biomedical stents, flexible electronics, smart actuators, and engineering structures, which attract extensive attention and possess a promising future in 4D printing metameral and dynamic covalent networks, as shown in Figure 3.

## 3. Mechanism

**Figure 4:**
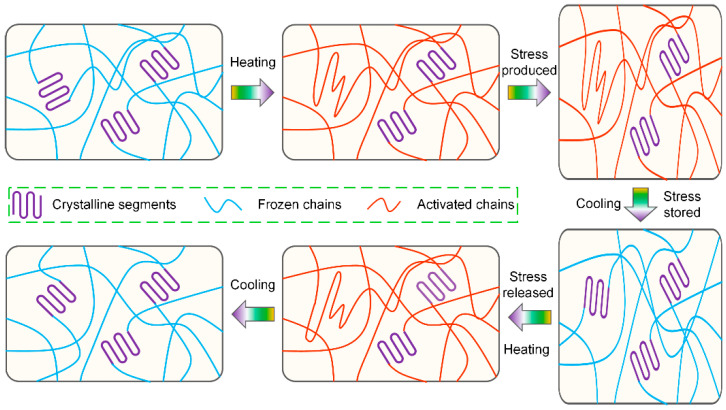
An illustration of the structural transition of heat-triggered thermoplastic SMPs during the shape memory process.

For thermoplastic SMPs, shape memory effect does not originate from the unique polymer chemistry reactions of structures, but is an integration of the molecular chains, reversible changes, and conformational entropy. To operate the shape memory effect, two structural components should be contained in SMPs matrix: permanent phases and reversible phases. The permanent phases provide stable structures for the storage/memory of permanent shape by preventing chain slippage/flow/creep upon deformation. In addition, the reversible phases could achieve the deformation or fixity of the temporary shape while being triggered by external stimuli through the motion of molecular chains, which could be accomplished through phase transitions, including glass–rubber transition (T_g_), crystallization/melting transition (T_m_), anisotropic/isotropic liquid crystal transition (T_LC_), or supramolecular association/disassociation [38,39]. In addition, the shape memory behaviors of thermoplastic SMPs commonly contain three stages: processing, programming, and recovery. Initially, the macroscopic structures could be fabricated during the forming process, which act as the original permanent shape. Subsequently, the temporary shape could be obtained through the external programmable force, upon external stimuli. Finally, the SMPs recover to the original shape while receiving the corresponding stimuli. During the deformation and recovery processes, the orientation of molecular chains occurs upon the applied force, where the stationary phases are conventionally the hard domains and the activated chains act as the switching domains, as shown in Figure 4.

**Figure 5:**
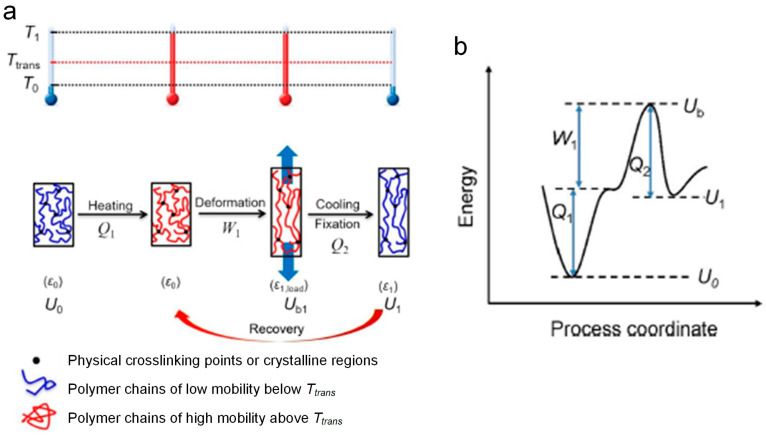
Shape memory mechanism of heat-triggered thermoplastic SMPs [40].

The heat-triggered thermoplastic SMPs act as an example for illustrating the shape memory mechanism, as depicted in Figure 5 [40]. At room temperature, the molecular chains in SMPs matrix are in the state of maximum entropy and minimum energy. At this time, SMPs exhibit the glass state with high strength and modulus. When heating the SMPs up to the transition temperature (T_trans_), the internal structures in polymer matrix are transformed and the motion of molecular chains is activated. SMPs are in the unstable state of high energy and low entropy, exhibiting the rubber state with low strength and modulus. Perform the external load onto SMPs for macroscopic deformation and maintain the load until cooling down the temperature. At this time, the active molecular chains are frozen, where the entropy and elastic potential energy are bound, and the internal stress is stored in polymer matrix. As a result, SMPs are fixed to the temporary shape. Afterwards, heat the SMPs up to the T_trans_ again, where the motion of molecular chains is actuated and the whole system is transformed from the state of high entropy and low energy to high energy and low entropy. The internal stress stored before is released for the actuation of the macroscopic shape recovery behaviors.

**Figure 6:**
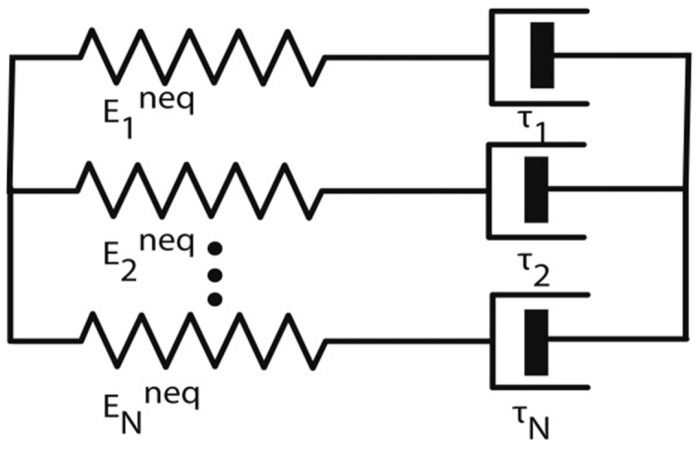
Multiple parallel Maxwell model [42].

In addition, the rheological model (parallel spring–dashpot model) could be used for the simulation of shape memory behaviors of thermoplastic SMPs [41]. The pattern consists of two parts, a spring (balanced branch) and a dashpot (unbalance branch) in parallel, and the significant change in dashpot relaxation time evaluates the shape memory properties of SMPs. In addition, a nonlinear viscoelastic model for the exploration of the physical mechanism behind shape memory behaviors is developed, as shown in Figure 6 [42]. For thermoplastic SMPs, physical entanglements are one of the domination elements for permanent shape, which could be evaluated by the viscoelastic relaxation behaviors with long relaxation time in the model. The parameter study indicates that the relaxation modulus can be used to predict the shape recovery performance of SMPs, in addition that the broad distribution of relaxation time, and the glass transition could adjust the shape memory behaviors.

Nevertheless, due to the linear chain structures, the shape memory properties of thermoplastic SMPs are usually not as good as those of thermoset SMPs. At present, there are two main approaches to improve shape memory performance: one is enhancing the elastic modulus of SMPs through the integration of reinforcing fillers, and the other is enhancing elastic strain energy through combing the SMPs matrix with the elastic materials [43,44].

## 4. SMPs Matrix Polymers

At present, thermoplastic SMPs matrix polymers have largely expanded, including polylactic acid, polyurethane, and polycaprolactone, in addition to the newly emerged materials, such as polynorbornene, liquid crystal elastomer, etc. In addition, plenty of biomaterials such as chitosan and lignin could be widely used as SMPs matrix polymers. Table 1 lists the commonly used thermoplastic SMPs matrix polymers. Various matrix materials reflect the extensive sources of raw materials, and develop diverse practical performance, which largely promote the development and utilizations of SMPs.

## 5. Diverse Stimuli Actuations

The development of heat-triggered thermoplastic SMPs is relative early, and the corresponding studies have been in-depth. Nevertheless, as peoples’ lives advance, it could be difficult for heat-triggered SMPs to satisfy the applications in certain scenarios and areas, which limits the development and applications of SMPs. Thus, the exploitation for the new-type actuation for SMPs has become research’s focus. At present, the main actuating responses are not only heat response, but also electrical response, magnetic response, light response, and solvent response [101,102].

### 5.1. Electrical Actuation

**Figure 7:**
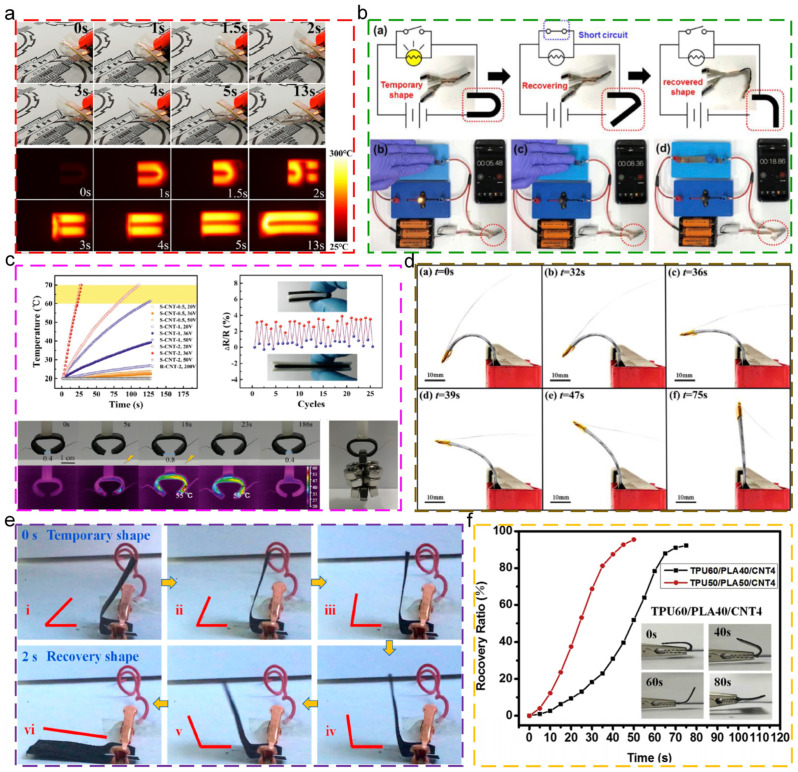
Electric-triggered shape memory behaviors: (**a**) digital photos and infrared thermal images during shape recovery process of electrical heater at 12V of applied voltage [103]; (**b**) reentrant composite as emergency circuit breaker under short circuit conditions [105]; (**c**) electric-triggered shape memory gripper [106]; (**d**) snapshots of shape recovery and temperature distribution of 4D-printed specimen during resistance heating [107]; (**e**) shape recovery process of conductive microfiber membrane at 30V [109]; (**f**) shape recovery ratio of TPU/PLA/CNTs composites as function of time [110].

Electrical triggered thermoplastic SMPs, as a kind of heat-triggered SMP through indirect heating, are composed by the conductive particles which could produce Joule heat upon the applied electric field and SMPs matrix materials. While the content of conductive particles reaches up to the threshold, the connected conductive networks are constructed in SMPs matrix. Upon the external voltage, the current generates in SMP systems. The conductive particles produce Joule heat and transform electrical energy into heat energy for heating the SMPs matrix. While the temperature reaches up to the T_trans_, the shape recovery behaviors are triggered. At present, the commonly used conductive particles are mainly metal particles [103] and carbon materials, including graphene [104], carbon nanotubes (CNTs) [105,106], carbon fibers [107], and carbon black [108]. Huang [103] embedded the conductive aluminum mesh into the shape memory PI film to prepare the flexible transparent electrical heater with rapid response and high operating temperature, as shown in Figure 7a. In addition, the electrical heater could actively deform due to the unique variable stiffness, and the high transition temperature of 230 °C largely extended the application scenario. Kang [105] used graphene–CNTs to fabricate the necked micro honeycomb structures for stretchable conductive devices, as depicted in Figure 7b. In addition, the shape memory PU was immersed into the honeycomb structures, achieving low resistance and excellent tensile strength for the composites. The regular distribution of graphene–CNTs imparted polyurethane in an adaptive environment for heterogeneous nucleation and crystallization growth, promoting the shape memory effect. Additionally, the “on-off” illustration of portable battery circuit indicated that the composites could be used as a circuit breaker. Xu [106] embedded the separated CNT conductive network into poly (ethylene-co-octene) matrix for the preparation of electrical triggered shape memory composites, as shown in Figure 7c. While the CNT content was 2 vol%, the conductivity was 0.046 S/cm. Thus, these composites could maintain excellent actuating performance during low driving voltage (below 36V). Additionally, the action illustration of electrical triggered gripper indicated that these composites could be widely used in fields of artificial muscle and bionic robot. Zeng [107] fabricated the continuous carbon fiber-reinforced shape memory PLA composites through the 4D printing technique, as depicted in Figure 7d. These composites exhibited excellent electrical triggered shape memory behaviors and electrothermal stability. In addition, the quantitative investigation for the bending angles and temperature of composites during the deformation process indicated that the real-time deformation of composites could be monitored through the measurement of resistance. Zhang [109] prepared the conductive shape memory PLA microfiber membranes through the electrospinning technique and chemical vapor polymerization. The prepared membranes exhibited excellent electrical triggered shape memory behaviors which could recover to the original shape within 2s at 30V, as shown in Figure 7e. Liu [110] fabricated the thermoplastic polyurethane (TPU)/PLA composites with the rapid electric-triggered shape memory behaviors through the integration of CNTs, as shown in Figure 7f. In addition, the composites exhibited great mechanical properties, processing capability, and low cost.

### 5.2. Magnetic Actuation

**Figure 8:**
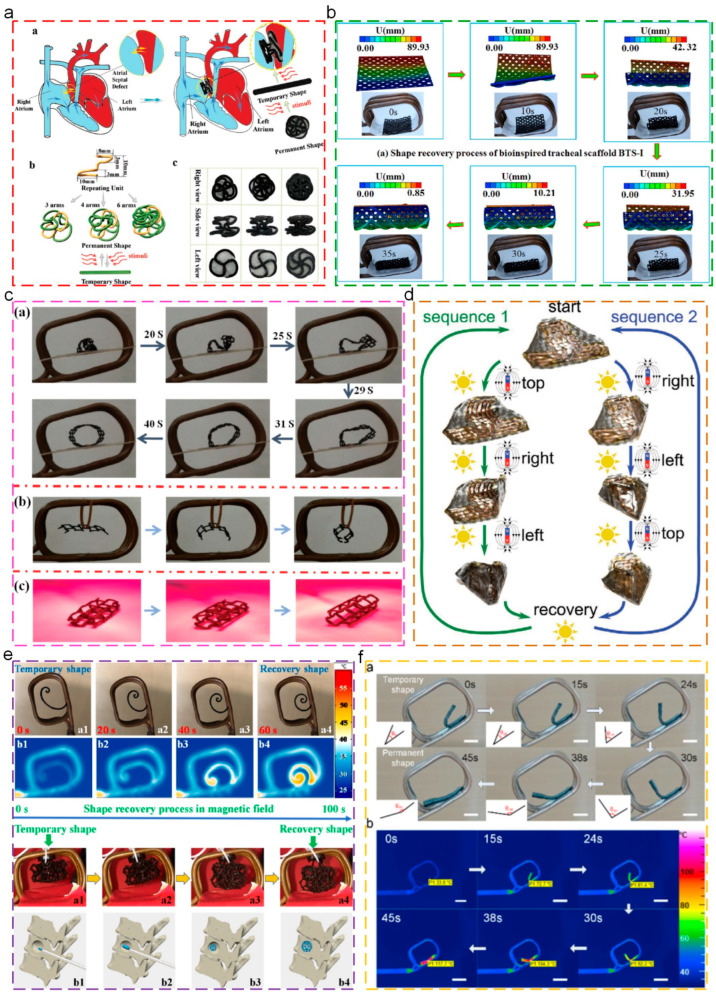
Magnetic-triggered shape memory behaviors: (**a**) schematic illustration of occlude before and after interventional therapy, in addition to design and samples of occluder frames with different arms [111]; (**b**) fast-transforming and shape locking of SMPs via superimposed magnetic fields [112]; (**c**) recovery process of 4D-printed shape memory PLA/Fe_3_O_4_ composite tracheal stent [113]; (**d**) illustration of two actuation sequences for assembly of magnetic origami without predefined hinges [114]; (**e**) magnetic field-triggered shape recovery behaviors of 4D-printed structures with 15% Fe_3_O_4_ at 27.5 kHz [117]; (**f**) magnetic-triggered shape memory behavior of PEEK/Fe_3_O_4_ composites [118].

Magnetic-triggered thermoplastic SMPs can operate shape recovery behaviors when experiencing external magnetic field, which are composed by magnetic response particles and SMPs matrix materials. Similarly to the electrical triggered SMPs, magnetic-triggered SMPs are also a kind of indirect heating SMP materials; nevertheless, the difference and advantage are mainly the remotely non-contact actuation, which largely extended applications in biomedical areas. At present, the commonly used magnetic response particles mainly include Fe_3_O_4_ [111,112,113], NdFeB [114,115], and Fe_2_O_3_ [116], which could absorb the magnetic energy upon the external alternating magnetic field and transform it into heat for SMPs matrix. While the temperature is increased up to the transition temperature, the SMPs spontaneously recover to the original shape. Lin [111] prepared a biodegradable customized shape memory occluder through the 4D printing technique, as depicted in Figure 8a. The integration of magnetic Fe_3_O_4_ particles into shape memory PLA matrix could achieve the remote controlling of the occluder after implantation. In addition, the excellent cytocompatibility and histocompatibility were conducive to cell adhesion and proliferation. Meanwhile, the customized shape memory occluder ensured ideal fitness and provided adequate support when encountering defects. Zhao [112] reported a kind of customized shape memory biological tracheal stent, as shown in Figure 8b. Compared with the conventional tracheal stent, SMP-based stents exhibited better fitness for the optimal fixity condition. Meanwhile, the integration of magnetic Fe_3_O_4_ particles imparted remote actuation. In addition, the stents based on glass sponge’s microstructure possessed high strength and stability, which could be suitable for the complex environment of soft tissue. Zhang [113] prepared the shape memory PLA-based tracheal stents through the 4D printing technique, as shown in Figure 8c. In addition, the integration of magnetocaloric Fe_3_O_4_ particles imparted excellent magnetic-triggered shape memory behaviors, including rapid recovery rate (within 90s) and high recovery ratio (over 99%). These 4D printing tracheal stents provided a strategy for implantable medical devices and minimally invasive surgery for biomedical applications. Ha [114] prepared magnetic origami structure actuators which could perceive the direction and displacement and monitor the self magnetization state, as depicted in Figure 8d. These magnetic-triggered actuators were composed by thermoplastic SMPs matrix materials (thickness of 60 μm) and NdFeB particles, which exhibited programmble folding and recovery behaviors, along with high strength and perception ability. Zhang [117] investigated the shape memory behaviors of the 4D-printed PLA/Fe_3_O_4_ composite structures, as shown in Figure 8e. In addition, the bone tissue-like structures were prepared, and the shape recovery behaviors could be triggered by the magnetic fields. During the shape recovery process, the surface temperature was about 40 °C, which was physiologically adaptive. Yang [118] synthesized the shape memory poly(ether ether ketone) (PEEK), and incorporated the magnetocaloric Fe_3_O_4_ particles for the achievement of remote magnetic-triggered shape memory behaviors (Fe_3_O_4_ content over 10 wt%), which exhibited potential in fields of biomedical applications, smart electronics, and aerospace structures, as shown in Figure 8f.

### 5.3. Light Actuation

**Figure 9:**
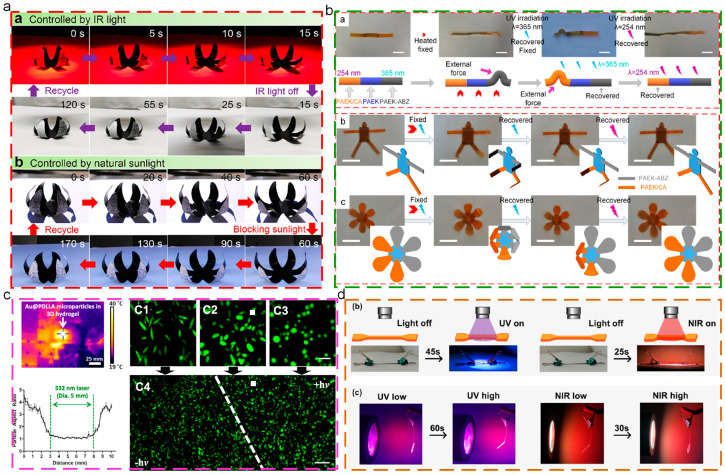
Light-triggered shape memory behaviors: (**a**) cyclic shape changes in bionic flower made of bilayer composites controlled by IR light and natural sunlight [128]; (**b**) wavelength selective responsive shape memory structures [129]; (**c**) shape memory is spatiotemporally controlled by laser irradiation [123]; (**d**) photo-responsive behavior of composites [125].

Light-triggered thermoplastic SMPs are able to achieve the shape recovery process upon experiencing external irradiation, exhibiting remote controlling, instantaneity, and spatial accuracy [119]. According to the light actuation mechanism, light-triggered SMPs could be divided into two categories: photothermal-triggered SMPs and photosensitive reaction-triggered SMPs. The former are composed of photothermal particles and SMPs matrix materials, and the essence is indirect heating SMPs. At present, the commonly used photothermal particles are mainly carbon black [120], graphene oxide [121], and Au nanorods [122]. Photosensitive reaction-triggered SMPs could make corresponding reactions (isomerization reaction, crosslinking, and de-crosslinking reaction) upon certain irradiation, due to the integration of photosensitive groups into SMPs matrix. During the process, the internal stress stored before could be released, and the whole SMPs recover to the original shape. At present, the reported photosensitive groups mainly include azobenzene [123,124,125], cinnamic acid [126], and spiropyran groups [127]. Cui [128] prepared the carbon nanotubes/chlorinated poly(propylene carbonate) composites through melt blending, which exhibited excellent light-triggered shape memory properties. In addition, the composite–paper bilayer films were prepared for the fabrication of an artificial flower, which could open under sunlight and close after blocking the sunlight, as shown in Figure 9a. Yang [129] constructed the light-triggered shape memory PAEK hybrid structures, which possessed the wavelength selective responsive shape memory behaviors upon the different wavelengths of irradiation. Moreover, the smart “man” and “flower” were fabricated to exhibit selective responsive actions, as shown in Figure 9b. Guo [123] prepared the light-triggered shape memory composite microspheres by encapsulating Au nanorods in PLA matrix. These smart microspheres could keep the anisotropic shape upon the body temperature, which, nevertheless, could recover to the original sphere shape while the temperature was slightly increased, at the range of 37–45 °C. Additionally, due to the photothermal effect of Au nanoparticles, these shape memory microspheres exhibited spatial control capability during shape recovery behaviors, as depicted in Figure 9c. Chen [125] prepared a new multifunctional programmable artificial muscle which combined the advantages of semi-crystalline polymers and liquid crystal elastomers, as shown in Figure 9d. These nanocomposites combined the enhanced performances of artificial muscle with programmability, in addition that the cycle deformations that could be achieved within 30 s due to the photoisomerization of azobenzene groups and photothermal effect of Au nanorods.

### 5.4. Solvent Actuation

**Figure 10:**
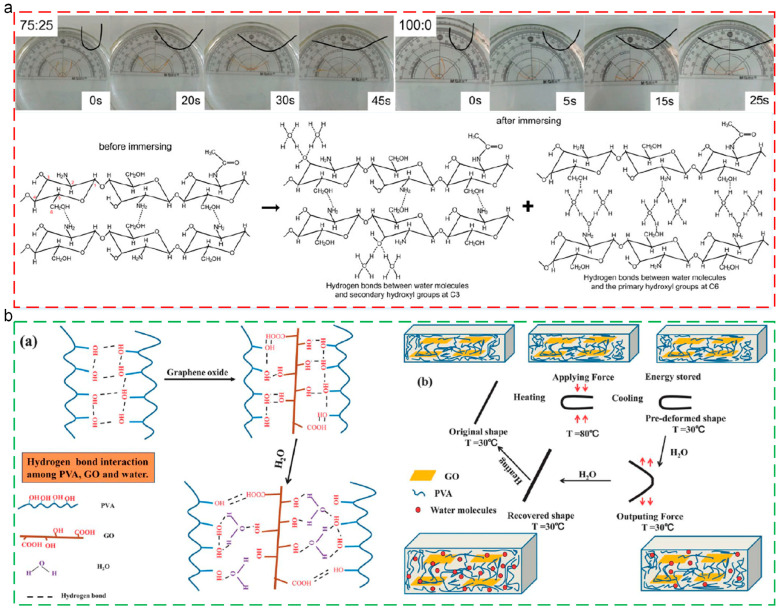
Solvent-triggered shape memory behaviors: (**a**) recovery process and mechanism of CS/GL composite film in various ratios of mixed water/ethanol solutions [132]; (**b**) illustration of hydrogen bonding interactions among PVA, GO, and water, and shape memory PVA/GO materials actuated by water [133].

Solvent-triggered thermoplastic SMPs are a kind of smart material which could be triggered by an external solvent. While the SMPs are immersed in solvent, the solvent molecules enter into the polymer molecular chains, enhancing the flexibility of SMPs. Meanwhile, the interaction between the solvent molecules and polymer molecular chains might disturb the previous non-covalent bonds among the polymer molecular chains. The two parts decreased the transition temperature of SMPs, for actuating the shape memory behaviors at low temperature. Water, as rich source, exhibits low cost and wide distribution, which could be an ideal solvent for the actuation of SMPs [130,131]. Zhang [132] prepared CS/glycerol (GL) composite film and investigated the water/ethanol mixed solvent-triggered shape memory behaviors. While the molar ratio of water molecules and ethanol molecules was over 2:1 in the mixed solvent, the water molecules could not only be combined with the ethanol molecules but also interacted with the CS molecules for forming hydrogen bonds. Thus, the stress stored in CS molecular chains before was released, and the shape recovery behaviors were triggered, as shown in Figure 10a. Qi [133] integrated graphene oxide (GO) into PVA matrix for the fabrication of water-triggered shape memory composites. The strong hydrogen bonding interactions between PVA and GO performed physical crosslinking points, which largely improved the shape memory properties. In addition, the water-triggered shape recovery behaviors could be obtained while the SMPs were immersed in water, demonstrating the plasticizing effect of water on PVA materials, as depicted in Figure 10b. These water-triggered PVA/GO composite materials provided a strategy for the fabrication of solvent-triggered SMPs.

**Figure 11:**
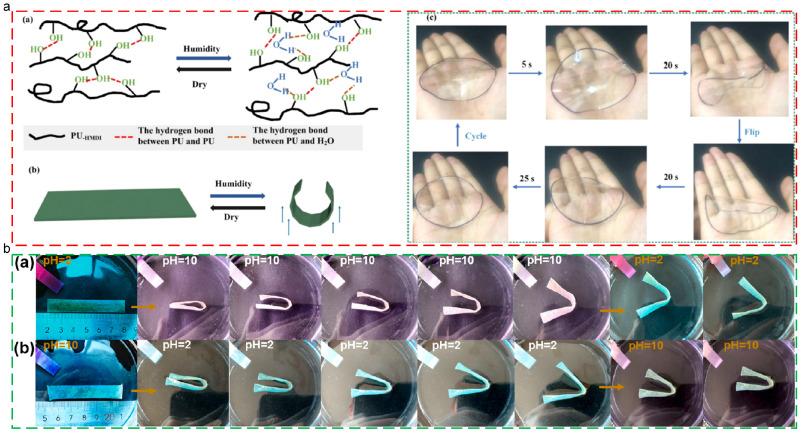
Moisture-triggered and pH-triggered shape memory polymers: (**a**) moisture-triggered shape memory polyurea; (**b**) shape memory process of TPU/CB/CNC-5 strips being immersed in (**a**) HCl solution (pH = 2) and (**b**) NaOH solution (pH = 10).

Despite the solvent actuations mentioned above, the shape memory effect of thermoplastic SMPs could be triggered by environmental moisture and the change in pH. Liu [134] presented a transparent, humidity-responsive shape memory polyurea with exceptional mechanical robustness and cryogenic flexibility, which achieved bioinspired hard-soft nanophase architecture and hierarchical hydrogen-bonded networks, as depicted in Figure 11a. The material exhibited zipper-like reversible bonding dynamics, where stress-responsive hydrogen bond dissociation and thermal re-bonding enabled humidity-actuated shape transformation. The synergistic combination of strong hydrogen-bonded hard segments and weakly bonded soft segments established a paradigm for designing high-performance stimuli-responsive polymers. Wu [135] developed shape memory nanocomposite films comprising thermoplastic polyurethane (TPU), carbomer (CB), and cellulose nanocrystals (CNCs). The films exhibited stimuli-responsive shape recovery under aqueous, ethanolic, thermal, and pH-variable conditions, with pH-dependent bond dynamics governing reversible structural transitions. While alkaline environments induced partial structural damage during recovery, acidic conditions restored mechanical integrity and enhanced load-bearing capacity, as shown in Figure 11b.

## 6. Applications

Due to the unique programmable deformation and recovery ability, in addition that the easy processing and recycling capacity, thermoplastic SMPs have attracted significant attention. Due to their multifunctionality and excellent performance, thermoplastic SMPs materials have been widely used in various aspects of industrial production and daily life, particularly in fields of biomedical stents, flexible electronic, smart actuators, and engineering structures.

### 6.1. Biomedical Stents

**Figure 12:**
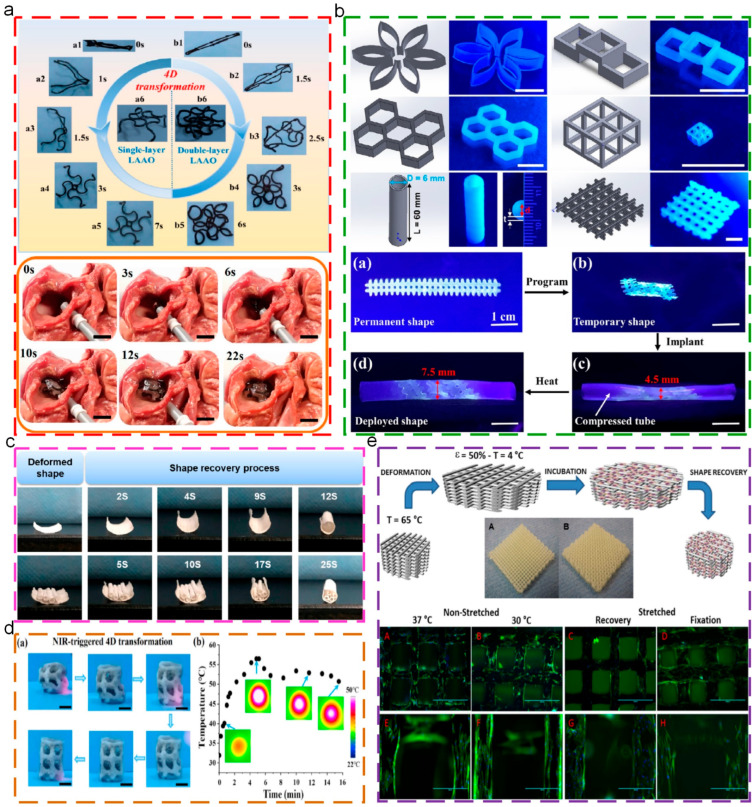
Applications of thermoplastic SMPs in biomedical stents: (**a**) shape memory process and feasibility demonstration of closure [136]; (**b**) 3D-printed structures and vascular stent [137]; (**c**) shape recovery process of tubes from their deformed temporary planar shapes to permanent tubular shapes [138]; (**d**) precisely controlled 4D transformation and temperature evolution of GO-functionalized PBS/PLA porous scaffold actuated by NIR laser [139]; (**e**) working principles behind 4D scaffolds, and Actin fibers (phalloidin, green) and nuclei (DAPI, blue) staining of dynamically seeded human mesenchymal stromal cells [140].

At present, SMPs have been widely used in biomedical applications, such as surgical suture, minimally invasive artificial stent implantation, and tissue repairing. Lin [136] used a 4D printing technique to prepare customized left atrial appendage occludes, as shown in Figure 12a, for matching tissue deformation and reducing complications. Through the iterative optimization of the stress–strain curves of left atrial appendage and left atrial appendage occludes, materials were obtained that possessed suitable mechanical performance for tissue. Meanwhile, the effect of the degradation on mechanical strength was evaluated via in vivo degradation tests. Additionally, the shape memory occludes exhibited excellent durability and biocompatibility. Zhang [137] used poly (glyceryl dodecanoate) acrylate (PGDA) with the transition temperature of 20–37 °C as the raw material to prepare the deformable structures through a 4D printing technique, as shown in Figure 12b. These structures exhibited excellent shape memory behaviors, including high shape fixity ratio (100%), recovery ratio (98%), stable cycle performance (over 100 times), and rapid recovery rate (within 0.4s). In addition, due to the phase transition of PGDA, the Young’s modulus of structures could reduce 5 times the size to be suitable for tissue, indicating their spatial and mechanical adaptability, which put forward a strategy for customized biomedicine and biological scaffolds. Inspired by the stimuli-responsive deformation of SMPs, Wang [138] constructed the self-formed multichannel nerve catheter using shape memory materials, as shown in Figure 12c. The original tubular shape could be obtained through high temperature processing, which could temporarily deform to the plane shape for the uniform distribution of loading cells. Additionally, the structures could recover to the original tubular shape while the temperature was 37 °C. These multichannel catheters could promote cell growth and sciatic nerve repair, exhibiting potential in peripheral nerve regeneration. Lin [139] investigated the mechanical properties and shape memory performances of 4D-printed polybutylene succinate (PBS)/PLA composite structures. Furthermore, the integration of graphene oxide exhibited attractive photothermal properties for the achievement of remote and accurate controlling of the transformation of porous scaffolds, which conquered the challenges conventional heat-triggered shape deformations experience, as depicted in Figure 12d. Hendrikson [140] prepared shape memory PU-based 4D scaffolds through additive manufacturing. Additionally, while the cells were seeded onto the scaffolds with the temporary shape, the original shape was recovered to elongate the cells and change the cells’ shape, as shown in Figure 12e.

### 6.2. Flexible Electronics

**Figure 13:**
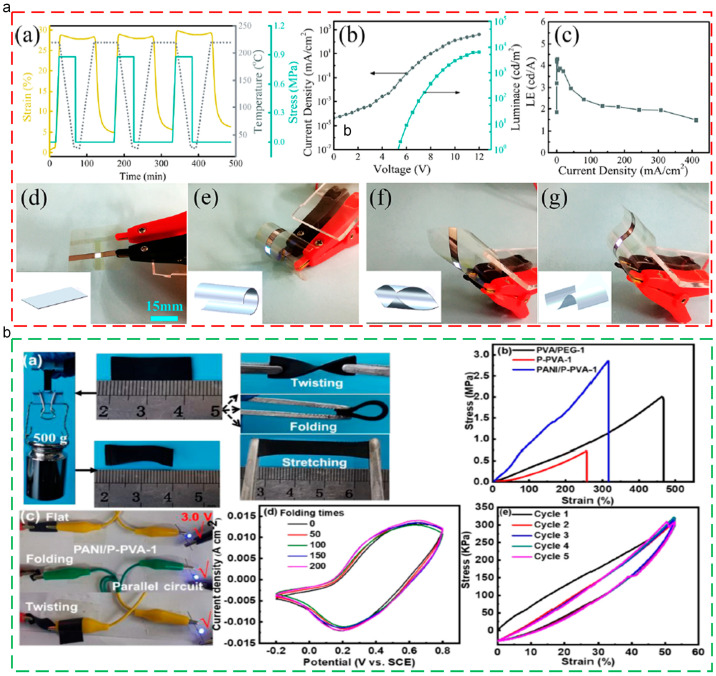
Applications of thermoplastic SMPs in flexible electronics: (**a**) shape memory ability of SMPI substrate and LEDs [142]; (**b**) photographs of elastic deformation and recovery of flexible electrode [143].

Flexible electronic technology is a new electronic technology where organic/inorganic electronic devices are loaded onto flexible/ductile substrates. Compared with the conventional electronic technology, flexible electronic devices possess better flexibility, which could deform to adapt to different operation environments [141,142,143,144]. In addition, due to the unique programmable deformation ability, using thermoplastic SMPs as the matrix materials for flexible electronic devices could largely improve environment adaptation and impart variable rigidity and active deformation capacity, which have gradually become the research’s focus. Huang [142] prepared flexible, transparent, and conductive shape memory PI composites. The colorless shape memory PI exhibited excellent optical transparency and heat resistance, which could be an ideal substitute for flexible electronic plates. Meanwhile, Au/Ag composite metal gate electrodes were embedded onto the SMPs through the self-cracking mold and solution coating for the achievement of a super smooth surface, excellent mechanical toughness and durability, strong adhesion, and excellent chemical stability. The light-emitting diodes based on the composites (LEDs) exhibited programmable deformation ability, which could be deformed from a 2D shape to a 3D shape. Meanwhile, the deformed 3D shape devices could recover to the original shape upon heating, exhibiting their value in smart optical electronic, as shown in Figure 13a. Du [143] fabricated the flexible polyaniline/PVA composite electrode, which exhibiting excellent shape memory ability and capacitance performances. The T_g_ of the composite was 75.9 °C, and the shape recovery behaviors could be finished within 10s at a temperature of 80 °C, as shown in Figure 13b.

### 6.3. Smart Actuators

**Figure 14:**
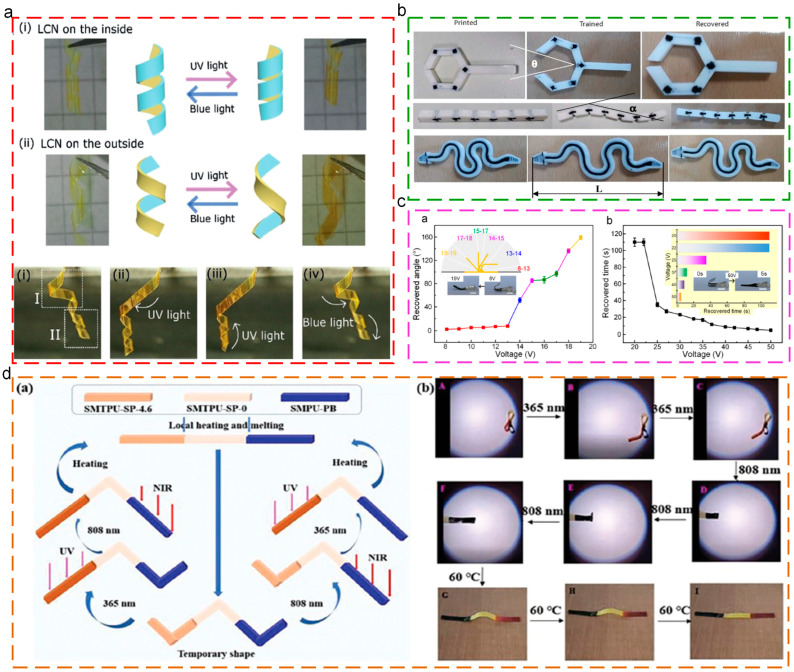
Applications of thermoplastic SMPs in smart actuators: (**a**) reprogramming actuation mode by shape configuration [145]; (**b**) shape memory actuation process [146]; (**c**) operation of voltage actuators: angle-mode and time-mode [147]; (**d**) diagram and photos of programmable multi-responsive multi-segments motion under 365 nm UV light, 808 nm NIR light irradiation, and 60 °C thermal field, respectively [148].

At present, due to the great deformation ability, controllability, and durability, smart actuators have been widely used in fields of engineering and biomedical applications. Verpaalen [145] prepared the light-triggered actuators through spray-coating the azobenzene-doped liquid crystal network (LCN) onto polyethylene terephthalate (PET). The original shape of the actuators could be customized, including origami-like folds or left- and right-handed helicity, as shown in Figure 14a. The shape recovery behaviors could be triggered by external light, such as winding, unwinding, and unfolding. Micalizzi [146] prepared shape memory actuators through the multi-material 3D printing technique. The integration of conductive filament imparted the electric-triggered shape recovery properties, as depicted in Figure 14b. Yang [147] integrated conductive CNTs into shape memory poly(aryl ether ketone) (PAEK) matrix for the fabrication of composite voltage actuators. The two kinds of operation modes (angle-mode and time-mode) were obtained for the different actuating behaviors upon different applied voltage, as shown in Figure 14c. Wang [148] grafted photosensitive spiropyran groups onto the shape memory polyurethane for the achievement of shape memory behaviors, photochromism, and mechanochromism. In addition, the selective actuation properties were fabricated for the operation of programmable regional selective response actions, exhibiting value for soft actuators, as shown in Figure 14d.

### 6.4. Engineering Structures

**Figure 15:**
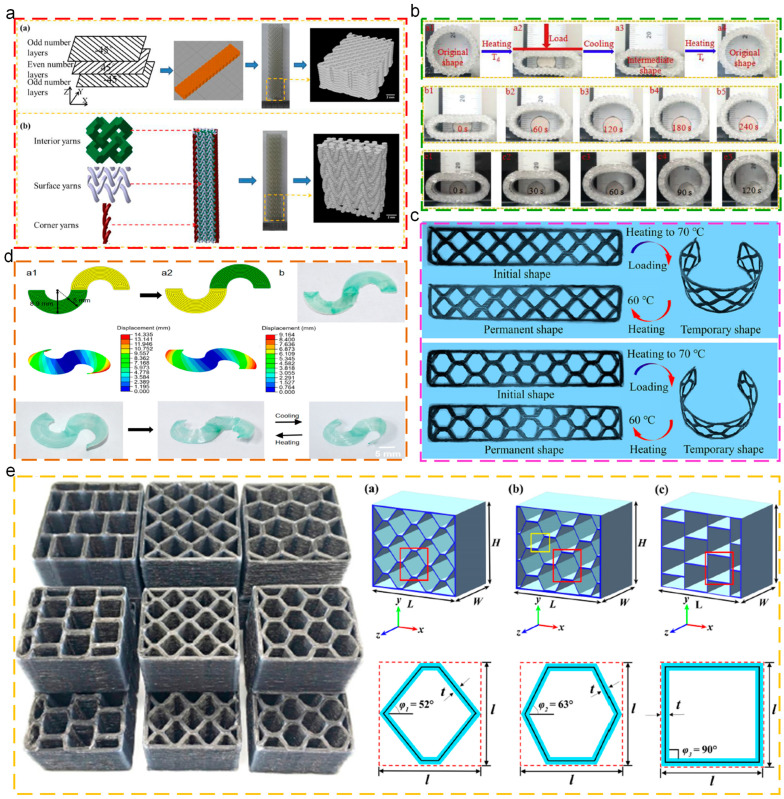
Applications of thermoplastic SMPs in engineering structures: (**a**) microstructural design and μ-CT imaging of 4D-printed preforms [149]; (**b**) shape memory cycle of 4D-printed circular braided tube [150]; (**c**) initial shapes, temporary shapes, and permanent shapes of 3D-printed CFRCTCSs [151]; (**d**) bidirectional bending reversible deformation of S-shaped semicircular ring model, which was filled with concentric circular printing path [152]; (**e**) prepared samples and geometric configurations of proposed honeycomb structures with various cells [153].

In engineering applications, low weight and high strength are dominant for structural materials. Meanwhile, low cost and easy processing are also significant. Thermoplastic SMPs, due to the intrinsic programmable deformation and re-processing abilities, exhibit potential for engineering structures. Liu [149] prepared silicone elastomer composites based on angle-ply laminated and rectangular braided preforms through the 4D printing technique, as depicted in Figure 15a. After integrating CNTs, the shape recovery behaviors could be more easily triggered, and the flexural load was enhanced. Zhang [150] prepared the 4D-printed circular braided tube polymers and the silicone elastomer composites, which exhibited enhanced shape memory force and high recovery force, as shown in Figure 15b. Zeng [151] prepared the continuous fiber-reinforced composite trapezoidal corrugated sandwich structures (CFRCTCSs) through the co-extrusion-based 3D printing technique. The bending properties and failure behaviors of structures were investigated through the three-point bending tests and analyzed by the theoretical models, which exhibited great consistency. In addition, CFRCTCSs exhibited excellent shape memory properties, which provided opportunities for lightweight structures in engineering systems, as depicted in Figure 15c. Wang [152] fabricated reversible deformable structures through the 4D printing technique using thermoplastic polyurethane and polylactic acid as raw materials. Moreover, diverse mathematical models were established to simulate the reversible deformation actions for composite laminates, as shown in Figure 15d. Zeng [153] prepared continuous fiber-reinforced composite honeycomb structures through the 4D printing technique (Figure 15e), which exhibited excellent shape memory properties. Additionally, these structures possessed better compression strength and specific energy absorption, indicating their potential as adjustable energy absorbing devices.

In addition, as for aerospace structures, the light weight of materials becomes the most significant factor for the structures. On the premise of ensuring the strength and stability of materials, reducing the quality as much as possible has become a technical difficulty that researchers urgently need to overcome [154]. Thermoplastic SMPs exhibit intrinsic light weight and easy processing and re-processing ability. Meanwhile, the enhanced strength and stability could satisfy the requirements, and the unique programmable deformation ability greatly expands the using for space deformable structures [155,156,157].

## 7. Future Directions

### 7.1. Four-Dimensional Printing Metamaterial

**Figure 16:**
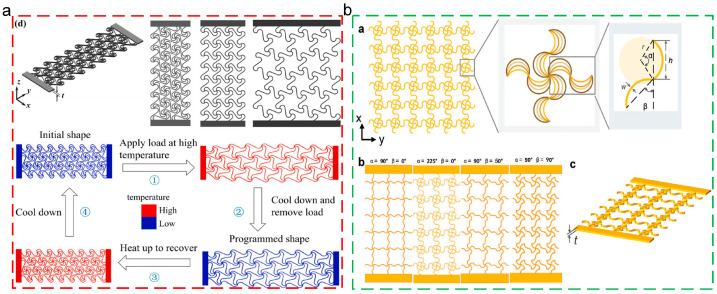
Four-dimensional printed metamaterial: (**a**) printed models of metamaterial and shape memory cycle [161]; (**b**) structural model and geometric parameters of mechanical metamaterial [164].

Thermoplastic SMPs, due to the linear molecular chain structures, exhibit excellent processing ability, including diverse processing methods (molding processing, extrusion molding, addictive manufacturing, etc.) and easy processing procedure. Addictive manufacturing (3D printing technique) could directly process the materials into three dimensional structures without any molds, satisfying the complicated and personalized requirements for structures and devices. Thus, smart deformable and self-adaptive structures could be fabricated using thermoplastic SMPs matrix polymers as raw materials, through the 3D printing technique, which could be named the 4D printing technique [158,159,160]. Metamaterial, fabricated by manual design, possesses unique physical and mechanical properties which do not occur in the natural materials, exhibiting potential in biomedical stents [161], smart devices [162], and flexible integrated devices [163]. Hence, using the 4D printing technique for the preparation of metamaterial-based structures has become the research’s focus. Wan [161] prepared programmable triangular, square, and honeycomb lattice metamaterial using the 4D printing technique, which exhibited large deformation and auxetic actions. In addition, due to the intrinsic shape memory properties, the metamaterial exhibited the tunable Poisson’s ratios and elastic moduli through regulating the topological parameters and temperature, as depicted in Figure 16a. Ren [164] used polyetheretherketone (PEEK) as the raw material to prepare metamaterial mesh structures through the 4D printing technique, as shown in Figure 16b. Due to the intrinsic shape memory properties, the programmability and re-configurability of metamaterial were obtained.

### 7.2. Dynamic Covalent Networks

**Figure 17:**
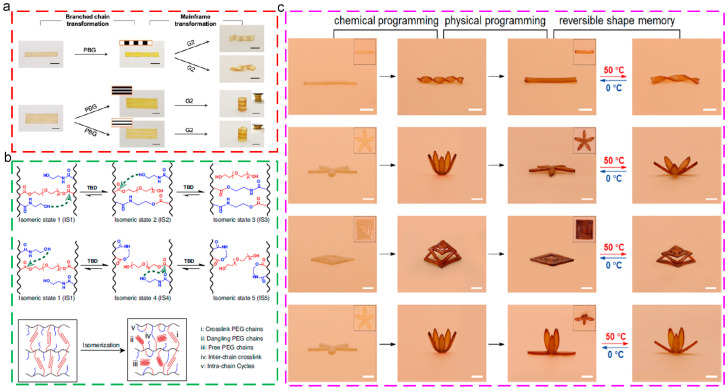
Dynamic covalent polymer materials: (**a**) on-demand programming of mechanical pattern and shape [168]; (**b**) design of dynamic covalent network and mechanism of topological isomerization [169]; (**c**) zero-set reversible shape memory via synergetic chemical and physical programming [170].

Generally, thermoplastic and thermoset are two independent polymer materials. The former exhibits excellent processing and re-processing ability; nevertheless, the latter possesses enhanced strength and performance. Interestingly, the occurrence of dynamic covalent networks, which could retain the advantages of thermosets yet be re-processed like thermoplastics, breaks the barrier between thermoplastic and thermoset, attracting plenty of attention [165,166,167]. These polymers usually exhibit thermosetting performance; nevertheless, the internal network topologies could be rearranged while the dynamical covalent bonds are activated. Therefore, these SMPs matrix polymers could exhibit the advantages of thermoset, including enhanced mechanical properties, stability, chemical resistance, and creep resistance, and those of thermoplastic, such as easy processing, re-processing, and recycle performances. Miao [168] prepared independently controlled macroscopic shapes and molecular architectures through the construction of dynamic covalent networks, which exhibited two different orthogonal topological transformations. These dynamic networks achieved the programmable shape and spatially definable mechanical properties, providing a strategy for the on-demand regulation of network polymers, as shown in Figure 17a. Miao [169] designed rearrangeable dynamic networks, which exhibited spatiotemporal regulation for manipulating the polymer properties, as shown in Figure 17b. In addition, the constructed shape memory polymers exhibited the designable multi-shape and reversible shape memory properties, extending the versatility for network polymers and possessing potential in fields of soft robotics, flexible electronics, and biomedical devices. Song [170] integrated transesterification catalyst into the polymer networks for the achievement of enhanced shape-shifting behaviors and reversible shape memory behaviors, as shown in Figure 17c.

## 8. Conclusions

Significant developments have been achieved for the multifunctionality and enhanced performance of thermoplastic SMPs, and the advanced applications have been largely expanded. In this paper, we introduced the mechanism of thermoplastic SMPs, in addition to demonstrating the matrix materials and applications. Furthermore, two promising potential development directions were proposed. Thermoplastic SMPs, due to the intrinsic linear molecular chain structures, possess apparent phase transition behaviors including glass–rubber transition and rubber-viscous flow transition, which are radically different from thermoset. Therefore, diverse processing methods, easy processing procedure, and recycling re-processing capability have been significant advantages for thermoplastic SMPs, expanding their application in fields of biomedical stents, flexible electronics, smart actuators, and engineering structures. In addition, with the emergence of the 4D printing technique, the easy processing thermoplastic SMPs exhibited tremendous value, which could be processed by addictive manufacturing. Four-dimensional printing metamaterial exhibited promising potential, which largely extends the fabrication and application of metamaterial structures with unique mechanical performance. Additionally, the occurrence of dynamic covalent networks broke the barrier between thermoplastic and thermoset, which combined processing ability with practical performance, promoting the applications of SMPs, in addition to their attractive performance such as self-adapting, self-healing, reshaping, and reversible deformations. We believe that better functionalized, portable, and controllable thermoplastic SMPs will emerge in the future. In particular, the recycle re-processing ability and enhanced practical performances will be assembled into the whole SMP materials.

## Figures and Tables

**Table 1:** The commonly used thermoplastic SMPs matrix materials.

Material		Transition Temperature (°C)	Feature	Application
Polylactic acid (PLA)	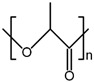	55−65	High strength, biocompatibility	Biomedical stents [45,46,47,48]
Polyurethane (PU)	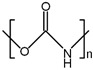	25−50	Body temperature actuation	Biomedical applications [49,50,51,52,53,54]
Polycaprolactone (PCL)		50–70	Functionality	Actuators [55,56,57,58]
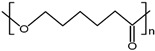				
Polyimide (PI)		220–250	Heat resistance	Engineering applications [59,60,61,62,63,64,65]
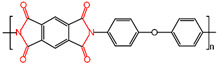				
Polyvinyl alcohol (PVA)	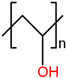	75−85	Water actuation	Actuators [66,67,68,69,70,71]
Polyolefin (PO)	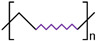	50−80	Low cost	Composite materials [72,73,74,75,76]
Polystyrene (PS)	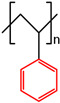	80−100	High strength	Composite materials [77,78,79]
Polynorbornene (PNBE)	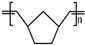	40−50	Deformation ability	Smart materials [80,81,82,83]
Ethylene vinyl acetate copolymer (EVA)		60–80	Humidity response	Biomedical applications [84,85,86]
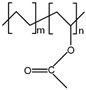				
Poly(aryl ether ketone) (PAEK)		130–150	Thermal stability	Aerospace applications [87,88,89,90,91,92]
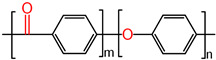				
Poly(methyl methacrylate) (PMMA)	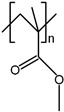	90−100	Optical transparent	Optical devices [93,94,95,96,97]
Chitosan (CS)		140–160	Biocompatibility	Biomedical applications [98,99,100]